# Erratum

**DOI:** 10.4269/ajtmh.2010.09-0257e

**Published:** 2010-03

In *Am J Trop Med Hyg 81(6):* 927–933 by Yadon and Schmunis, there is an error in reference 3. The correct reference is “Carlier Y, Torrico F, 2003. Congenital infection with *Trypanosoma cruzi:* from mechanisms of transmission to strategies for diagnosis and control. *Revista da Sociedade Brasileira de Medicina Tropical 36:* 767–771.” The journal regrets the error.

